# Introducing Attribute Association Graphs to Facilitate Medical Data Exploration: Development and Evaluation Using Epidemiological Study Data

**DOI:** 10.2196/49865

**Published:** 2024-07-24

**Authors:** Louis Bellmann, Alexander Johannes Wiederhold, Leona Trübe, Raphael Twerenbold, Frank Ückert, Karl Gottfried

**Affiliations:** 1 Institute for Applied Medical Informatics University Medical Center Hamburg-Eppendorf Hamburg Germany; 2 Department of Cardiology University Heart & Vascular Center Hamburg University Medical Center Hamburg-Eppendorf Hamburg Germany; 3 German Center for Cardiovascular Research (DZHK) Partner Site Hamburg-Kiel-Lübeck Hamburg Germany; 4 University Center of Cardiovascular Science University Heart & Vascular Center Hamburg University Medical Center Hamburg-Eppendorf Hamburg Germany

**Keywords:** data exploration, cohort studies, data visualization, big data, statistical models, medical knowledge, data analysis, cardiovascular diseases, usability

## Abstract

**Background:**

Interpretability and intuitive visualization facilitate medical knowledge generation through big data. In addition, robustness to high-dimensional and missing data is a requirement for statistical approaches in the medical domain. A method tailored to the needs of physicians must meet all the abovementioned criteria.

**Objective:**

This study aims to develop an accessible tool for visual data exploration without the need for programming knowledge, adjusting complex parameterizations, or handling missing data. We sought to use statistical analysis using the setting of disease and control cohorts familiar to clinical researchers. We aimed to guide the user by identifying and highlighting data patterns associated with disease and reveal relations between attributes within the data set.

**Methods:**

We introduce the attribute association graph, a novel graph structure designed for visual data exploration using robust statistical metrics. The nodes capture frequencies of participant attributes in disease and control cohorts as well as deviations between groups. The edges represent conditional relations between attributes. The graph is visualized using the Neo4j (Neo4j, Inc) data platform and can be interactively explored without the need for technical knowledge. Nodes with high deviations between cohorts and edges of noticeable conditional relationship are highlighted to guide the user during the exploration. The graph is accompanied by a dashboard visualizing variable distributions. For evaluation, we applied the graph and dashboard to the Hamburg City Health Study data set, a large cohort study conducted in the city of Hamburg, Germany. All data structures can be accessed freely by researchers, physicians, and patients. In addition, we developed a user test conducted with physicians incorporating the System Usability Scale, individual questions, and user tasks.

**Results:**

We evaluated the attribute association graph and dashboard through an exemplary data analysis of participants with a general cardiovascular disease in the Hamburg City Health Study data set. All results extracted from the graph structure and dashboard are in accordance with findings from the literature, except for unusually low cholesterol levels in participants with cardiovascular disease, which could be induced by medication. In addition, 95% CIs of Pearson correlation coefficients were calculated for all associations identified during the data analysis, confirming the results. In addition, a user test with 10 physicians assessing the usability of the proposed methods was conducted. A System Usability Scale score of 70.5% and average successful task completion of 81.4% were reported.

**Conclusions:**

The proposed attribute association graph and dashboard enable intuitive visual data exploration. They are robust to high-dimensional as well as missing data and require no parameterization. The usability for clinicians was confirmed via a user test, and the validity of the statistical results was confirmed by associations known from literature and standard statistical inference.

## Introduction

The amount and availability of data around us are constantly increasing. Researchers are increasingly using statistical models to guide their data-driven scientific work. However, as the relationships discovered increase in complexity, the models themselves are becoming gradually less transparent. In high-stake decision fields, such as health care, data explanation and justification of decision-making are essential for the applicability and distribution of novel technologies. Here, we present new methods for extracting statistical insights from large data sources and visualizing the results based on graph structures. The methods balance complexity and comprehensive description of the results on the one hand and clarity and interpretability for clinicians and patients on the other hand.

The availability of large quantities of medical data is growing [1,2] and thus enabling machine learning methods to play an ever-increasing role in medical research [3-5]. With the undoubtedly numerous advantages of “big data” in medicine arises the problem of increasing complexity and lack of transparency for clinicians [6,7]. In this context, the call for more interpretable statistical models is gaining more attention [8,9]. In addition to the interpretability of the applied models and results, good data visualization methods are key for the knowledge communication with clinicians and patients. Many methods have been developed over the years [10-12].

For data-driven analysis, approaches originating from the mathematical field of graph theory gain an increasing amount of attention for health care applications [13]. A graph consists of nodes representing arbitrary objects and edges each connecting 2 nodes corresponding to some form of relation between them. Graph-based database technologies, such as Neo4j (Neo4j, Inc) [14], allow more efficient retrieval of large amounts of data compared to traditional relational database systems [15,16], and many software tools for interactive, graphical user interfaces are available [14,17-20].

Knowledge graphs are a form of data representation capturing large quantities of data from potentially multiple sources in a graph structure. Existing data are usually processed and jointly represented to enable accessible, often visual, exploration of condensed knowledge across different data modalities and sources. Owing to their intuitive and versatile character, knowledge graphs have many applications in the medical domain [21]. Examples are the representation of biomolecular pathways [22], research related to COVID-19 or diabetes [23,24], knowledge about dietary supplement [25], and networks of complex disease interactions [26].

Statistical analysis discovering relations between variables within a medical data set can be captured within a graph structure. In this context, Bayesian networks are of increasing interest in the medical domain [27,28]. They represent conditional dependencies as edges and the absence of an edge as probabilistic independence [29]. Using these conditional dependencies, Bayesian networks can be used for inferring neural networks [30] or diagnosis prediction [31]. However, they are sensitive to missing data during the model training process [32]. Markov models describe states, for example, events during a patient’s hospital stay, as nodes and transition probabilities between states as edges [33]. As a result, Markov models are applied for the analysis of time-dependent dynamic processes in health care [33-35]. In association rule learning, relations between variables are extracted from a data set based on different measurements of interest, for example, conditional probability [36]. This concept is applied to extract patterns from clinical databases [37] or find suitable drug treatments [38]. All 3 approaches capture variable relations across a complete data set.

In this work, we developed the attribute association graph (AAG), a new graph structure capturing statistical knowledge extracted from a data set. We aimed to combine the focus of knowledge graphs on interpretability, accessibility, and visual exploration with graph-based statistical methods. We sought to develop a novel and robust tool for statistical analysis that is intuitively usable by physicians. We tailored our approach specifically to the needs of data-driven analysis in the medical domain by incorporating disease and control cohorts and aiming for robustness to high-dimensional or not normally distributed data, small sample sizes, and missing values. The graph is visualized, and nodes and edges representing variable relations of interest are highlighted to attract the attention of the user and facilitate the data analysis. We complemented the AAG with a dashboard for further data exploration. Only mouse clicking and search bar prompting in English are required for the navigation of the graph and dashboard. We aimed to evaluate the validity of the statistical analysis represented by the graph structure and dashboard. Therefore, we conducted an exemplary data analysis based on a large epidemiological study. The results of the analysis were compared with findings from literature and standard statistical inference using CIs of Pearson correlation coefficients. In addition, we assessed the usability of the visualization for medical researchers. We conducted user tests with physicians using standardized usability tests, user tasks, open feedback questions, and a free data exploration. The generated graph structure and dashboard are freely available to clinical researchers for exploration on their own computers.

## Methods

### AAG Definition

Our goal is to visualize participant attributes and the statistical traits and relationships between them in a compact, interpretable, and intuitive way. As a participant attribute, we consider a singular value or semantically meaningful value group for a variable, for example, “the participant was diagnosed with hypertension” or “participant has total cholesterol level above 200 mg/dL.” For the statistical analysis, we use simple metrics, which were found to be intuitive for clinicians [39]. The metrics are calculated for a disease and control group and compared to identify attributes with a large deviation. Thus, in contrast to traditional association rule mining [36], Bayesian networks [29], or Markov models [34], attributes can be selected, which appear more often in the disease group compared to the control group. As we analyze relations of singular attributes instead of associations between variables, our results are methodologically different from correlation analysis, such as chi-square tests [40] or Pearson correlation coefficients [41].

In the AAG, single attributes are captured as nodes and visualized as colored spheres of different sizes. Each node has parameters for the name of the attribute’s variable, its value, and a short description including units of measurement for metric variables. In addition, we assigned labels to each node depending on the broad categories of the represented attribute, for example, *Cardiac*, *Condition*, or *Medical History*.

For metric variables, we calculated reference ranges based on their value distribution within the whole data set. We defined the reference range as all values within SD around the mean. On the basis of reference ranges, we derived 3 additional nodes for the attribute associated with values below, within, and above the reference range. The 3 nodes inherit the parameter’s *name* and *description* from the original nodes. They have the value *low*, *normal,* or *high*. In addition, they contain the lower and upper bound of the reference range. All participants are assigned to 1 of the 3 nodes based on their attribute value. Thus, metric values, for example, patient laboratory results, are labeled in comparison to the whole data set and enriched with semantics.

In addition, we enriched the nodes with several statistical measurements of the described participant attribute within the data set. The resulting parameters are given in Table 1. Note that the relative attribute share accounts for the common problem of missing data [42,43] and is an upper bound to the relative total share. By measuring the difference and quotient of relative attribute shares, the distinction in attribute distribution between the 2 groups is expressed. The size and color of the node visualization capture parts of these measurements to support the data exploration with visual highlights.

**Table 1:** Statistical parameters for a node describing attribute *a* together with a short description and formula^a^.

Parameter	Description	Formula
Absolute count	Number of group members having attribute *a*	*c_i_*
Relative total share	Fraction of group members have attribute *a*	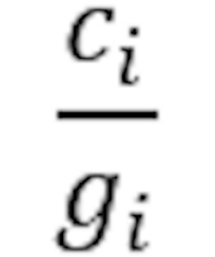
Relative attribute share	Relative total share, missing value adjusted	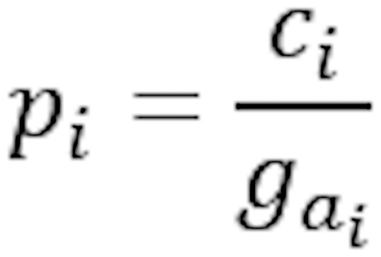
Relative attribute share difference	Absolute difference of relative attribute shares	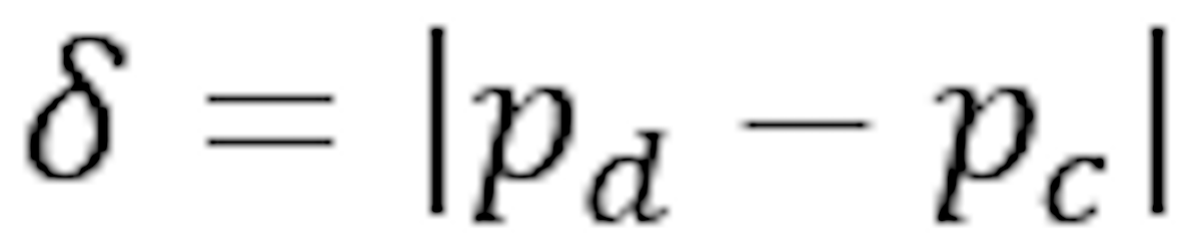
Relative attribute share quotient	Fraction of maximum and minimum relative attribute share	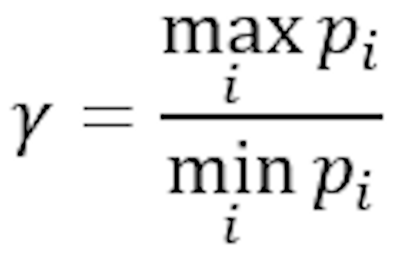

^a^Parameters with subscript *d* refer to the disease group. Parameters with subscript *c* refer to the control group. Subscript *i* refers to a definition for both groups, that is, *i*∈{*d*,*c*}. Let *g_i_* be the group size, and 
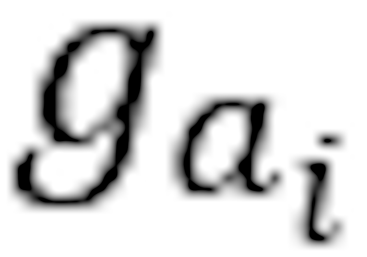
 be the number of group members having a valid value for the attribute *a*, that is, not a missing value.

We assigned a frequency label impacting the node’s size based on the maximum relative attribute share. Therefore, a node’s size indicates how common an attribute is within one of the groups. Let *p* be the maximum relative attribute share of a node. The node is assigned to 1 of the following 3 frequency label types:

- *p*≥0.5: labeled as *highly frequent*; the node has the largest size.
- 0.1≤*p*<0.5: labeled as *frequent*; the node has a medium size.
- *p*<0.1: labeled as *infrequent*; the node has the smallest size.

In addition, we assigned a distinction label to each node from which its color is derived. The distinction label, and thus the node color, indicates how much the attribute distribution differs between groups. Here, brighter colors signal a larger distinction. We reuse the symbols in Table 1. Each node is assigned 1 of 5 colors and distinction label types:

- δ≥0.2 or γ≥2.0:
  *p*_d_>*p*_c_: labeled as *highly related*; the node is colored in red.
  *p*_d_<*p*_c_: labeled as *highly inverse*; the node is colored in blue.
- (δ≥0.1 or γ≥1.5) and δ<0.2 and γ<2.0:
  *p*_d_>*p*_c_: labeled as *related*; the node is colored in orange.
  *p*_d_<*p*_c_: labeled as *inverse*; the node is colored in turquoise.
- δ<0.1 and γ<1.5: labeled as *unrelated*; the node is colored in beige.

Combining size and color, nodes that are displayed largest and brightest represent attributes with high frequency and large distinction between groups. As all parameters calculated for an individual node depend only on data for a single variable, the computation time needed for the calculation of all nodes of the graph scales linearly with the number of variables and linear with the sample size.

In the AAG, edges point from a source node to a target node, indicating the conditional dependence of the target attribute on the source attribute. The edges are displayed as lines with arrows pointing from the source node sphere to the target node sphere. The calculated statistical parameters for the conditional dependence are presented in Table 2. Note that the relative conditional share is conceptually equivalent to confidence in association rule learning [36]. By measuring the difference and quotient of the relative conditional share and the unconditional relative attribute share of the target node, the impact of the added condition is expressed. This impact can be negative if the unconditional relative attribute share is larger than the relative conditional share. We assign a type to each edge to capture the impact of the added condition. In the visualization, the line thickness of the edge is given by its type. We reuse the symbols in Table 2. Each node is assigned to 1 of the following 3 types:

- δ'≥0.2 or γ'≥2.0: assigned to the *high conditional difference* type; the edge has the thickest line.
- (δ'≥0.1 or γ'≥1.5) and δ'<0.2 and γ'<2.0: assigned to the *medium conditional difference* type; the edge has a thinner line.
- δ'<0.1 and γ'<1.5: assigned to the *low conditional difference* type; the edge has the thinnest line.

**Table 2:** Statistical parameters for an edge pointing from a source node *x* to a target node y^a^.

Parameter	Description	Formula
Absolute cooccurrence	Number of group members having both attributes of *x* and *y*	*o_i_*
Relative conditional share	Fraction of group members with attribute of *x*, also having attribute of *y*	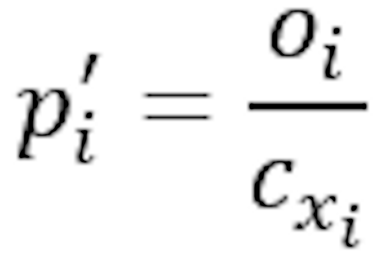
Conditional and unconditional target share difference	Absolute increase of relative conditional share compared to relative attribute share of *y*	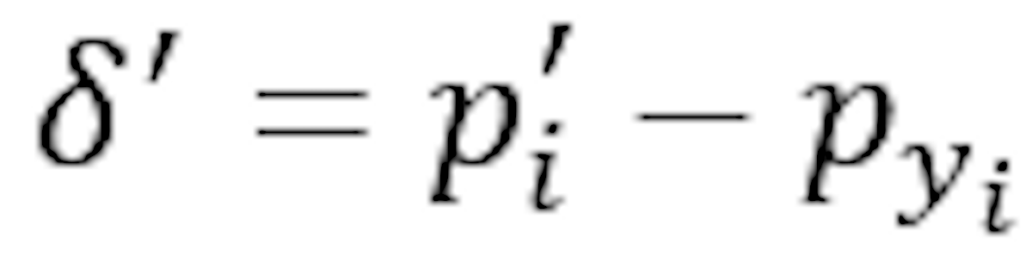
Conditional and unconditional target share quotient	Quotient of relative conditional share and relative attribute share of *y*	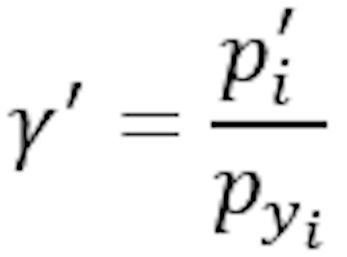

^a^Subscript *i* refers to a definition for both groups. Let 
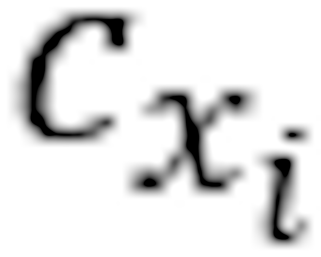
 be the absolute count of *x* and 
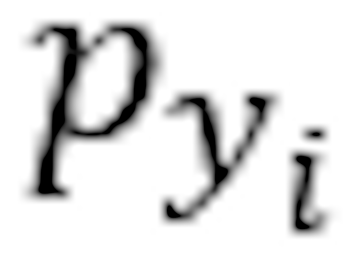
 be the relative attribute share of *y*.

The computation time for the generation of all the AAG’s edges scales quadratically with the number of variables in the data set and linear with the sample size.

In the last step, the nodes and edges are filtered by their statistical parameters to highlight the most relevant attributes and conditional dependencies. A detailed description of the filtering procedure is provided in Multimedia Appendix 1 [41,44,45]. We represented the extracted data in a graph structure using the graph data platform Neo4j [14] and the graphical user interface Neo4j Bloom (Neo4j, Inc) [19]. The graph structure can be navigated by mouse clicking and via a search bar typing prompts in English.

**Figure 1:**
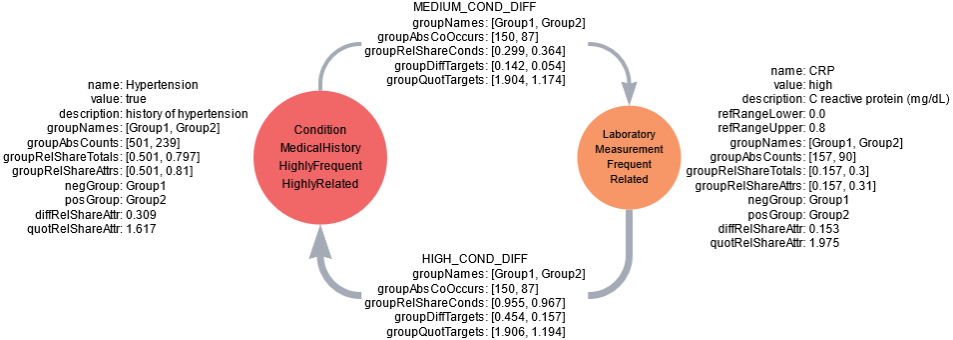
An attribute association graph with 2 nodes represented as spheres and 2 edges represented as lines with arrows. The arrow indicates the target node of the edge. Node parameters are depicted next to the spheres. Labels are shown inside the spheres with one label per line. The edge’s parameters are depicted on top of the edge. The heading above the edge’s parameters specifies the edge type (MEDIUM_COND_DIFF for medium conditional difference, HIGH_COND_DIFF for high conditional difference). Absolute counts (groupAbsCounts), relative total shares (groupRelShareTotals), relative attribute shares (groupRelShareAttrs), difference between relative attribute shares (diffRelShareAttr), quotient between relative attribute shares (quotRelShareAttr), absolute cooccurrence (groupAbsCoOccurs), relative conditional share (groupRelShareConds), difference to target relative attribute share (groupDiffTargets), and quotient to target relative attribute share (groupQuotTargets) are depicted as lists with the score for group 1, followed by the score for group 2. Group 2 is the disease group (posGroup), and group 1 is the control group (negGroup). The color of the sphere indicates the deviation label of the node: orange (related) and red (highly related). The size of the sphere indicates the frequency label from medium (frequent) to the largest size (highly frequent). The line thickness indicates the type of edge from medium (medium conditional difference) to thickest (high conditional difference). Descriptions of all parameter names, edge types, labels as well as color, size and thickness encoding can be found in the ZFDM repository. CRP: C-reactive protein.

Figure 1 [46] shows a minimal fictional example of an AAG with 2 nodes capturing fictional data about history of hypertension and high C-reactive protein (CRP) measurements as well as their relationship in participant group 1 (control group) and 2 (disease group). We conducted a hypothetical data analysis, as we intend the AAG to be used. For CRP measurements (mg/dL), a fictional reference range of 0.0-0.8 was derived. From the difference of the relative total share and relative attribute share, we can infer existing missing values on group 2 for both attributes. In group 1, no missing values exist because relative total share and relative attribute share do not differ. Regarding the quotient of relative attribute shares, we can infer group 2’s participants being almost twice as likely to show a high CRP value. Thus, a CRP measurement >0.8 mg/dL might be highly related to the condition or property of group 2 compared to participants of group 1. A history of hypertension appears approximately 30% more often in group 2, giving a 60% proportional increase. As a result, its node is labeled as highly related to the condition or property of group 2. Viewing the data of the edges, we find that almost all participants with a high CRP measurement also have a history of hypertension in both groups. Therefore, high CRP values could be an indicator for hypertension in both fictional groups. Conversely, only approximately one-third of participants with a history of hypertension also show high measurements of CRP. This pattern of conditional relationship is similar between groups and could thus be independent of the group definitions, for example, medical condition and control group.

### Dashboard

To complement the AAG, we generated a dashboard using the NeoDash (Neo4j, Inc) [17] toolkit. With the dashboard, users can investigate the average and distribution of metric variables across participant groups in more detail. In addition to the cardiovascular disease and control cohorts, the group of all participants contained in the Hamburg City Health Study (HCHS) data set was included. We developed 2 different tabs. The first tab allows for comparison of participant groups. We included the sizes of disease and control group. In addition, variable distributions can be compared between groups. For this purpose, we applied the following workflow to all metric variables and participant groups. First, we measured the variable average within the group. Second, we generated a binned distribution by rounding the measurements to multiples of 0.1, 0.5, 1, 5, 10, or 50 depending on the SD within the group. Bins containing <0.5% of the participants or <3 participants are summarized. We removed distributions without any bins fulfilling these requirements. The user can select 2 groups and variables for the distributions shown in the first tab of the dashboard. The averages of all metric variables for all 3 groups are shown in the first tab as well. To make them comparable in a figure, the averages of each variable are normalized by the maximum average of that variable. In the second tab, the user can investigate the relationship between 2 variables within a participant group. For the first variable, the generated binned distribution across the group is shown. For the second variable, we use precalculated averages of participants within a bin. The x-axis of the resulting figure shows the bin values of the first variable, and the y-axis shows the average value of the second variable for participants of that bin.

### HCHS Data Set and Cohort Selection

To evaluate the AAG and dashboard, we used an exemplary data exploration workflow of a large epidemiological cohort study. We compared the results with findings from literature and standard statistical analysis. The HCHS is a single-center, prospective, observational, population-based cohort study of 45,000 randomly selected residents of the metropolitan region of Hamburg, Germany, aged between 45 and 74 years. The study design has been published [47], and the study is registered [48]. The study focuses on major chronic diseases, causes for their development, as well as factors for survival and support for life in survivorship. The study considers >6000 properties per participant. The data are raised from 18 examinations, primarily targeting major organ systems, as well as questionnaires about medical and family history, physical condition, dietary habits, lifestyle, and various other topics. The examinations will be repeated after 6 years to obtain large-scale, long-term assessments. For this analysis, the HCHS committee provided a subset of the whole HCHS data set focusing on cardiovascular and cancer diseases. The subset consists of 524 selected attributes for the first 10,000 participants enrolled in HCHS, including information about laboratory analyses; electrocardiography (ECG); magnetic resonance imaging; vascular ultrasound examinations; blood pressure measurements; cardiovascular and cancer medical history questionnaires; as well as dietary, lifestyle and sleeping habits. We selected 131 (25%) of these 524 attributes, translated their descriptions to English, assigned labels to each variable to broad variable groups, and added Systematized Nomenclature of Medicine Clinical Terms [49] or Logical Observation Identifier Names and Codes [50] codes. When no directly fitting code was found, we chose the code of a related term. A full list of all variables, descriptions, labels, vocabulary codes, and data types can be freely accessed [46]. In some cases, the reference ranges calculated for the AAG deviated from the usual reference ranges known from the literature because of a different value distribution in the HCHS data set. In these cases, we manually adjusted the reference intervals according to the Merck Manual of Diagnosis and Therapy manual [44]. A full list of the adjusted reference ranges can be found in Table S1 in Multimedia Appendix 1. In this work, we focused on participants with a general cardiovascular condition. We included participants in this cohort who met any of the following criteria: showed any pathological cardiovascular findings during the cardiac magnetic resonance imaging examination; had a missing sinus rhythm; had a finding of atrial fibrillation or flutter in the ECG check; or reported a medical history of cardiac infarction, coronary artery disease, angina pectoris, congestive heart failure, myocarditis, or valvular endocarditis in the questionnaire. As a result, the disease cohort contained 1917 participants. In addition, we derived the control group of 8083 participants not exhibiting any of the conditions and findings.

### User Tests

#### Study Design

We conducted a user test using a mixed methods approach to evaluate the usability of the AAG. The associated questionnaire can be found in Multimedia Appendix 2. We did not consider the proposed dashboard in the user test, as dashboards are widely used in the medical domain [11,51-53]. The usability testing consisted of 3 main parts in the following order: (1) in a 30-minute preparation phase, participants independently worked through the AAG user manual and the Neo4j Bloom overview website [19]; (2) a semistructured interview with open feedback questions and user tasks was conducted; and (3) participants completed the System Usability Scale (SUS) [54]. The SUS is a standardized and validated instrument for usability testing of systems, frequently used in this context [25,52,55,56]. The SUS comprises 10 questions rated on a 5-point Likert scale. The total score, ranging from 0 to 100, is calculated from all questions to ensure comparability. With the addition of user tasks and feedback questions tailored to the AAG, we aimed to create additional insights on the usability of the specific parts of the graph as well as observe the data exploration conducted by the users. The user tasks can be grouped into three categories: (1) reproducing the introduced labels and metric parameters; (2) using the application functionalities necessary for exploration; and (3) conducting a free exploration of 2 AAG subgraphs of the HCHS data set: first, the 10 nodes with the highest quotient of relative attribute shares related to the cardiovascular disease group; and second, the subgraph of nodes regarding laboratory measurements. The user results for tasks of categories 1 and 2 were evaluated as correct or incorrect by the authors. During the exploration of the 2 subgraphs, the users were asked to verbalize their findings, and the results were recorded and categorized by the authors. The participant answers to the open feedback questions were also broadly categorized by the authors.

#### Participant Recruitment

The study participants for the user tests included 10 physicians from various specialties and fields of activity. This group comprised 2 anesthetists, 2 cardiologists, 1 neurologist, 1 radiologist, 2 resident doctors in the field of child and adolescent psychiatry, 1 medical student in the final year, and 1 physician working in the public health sector. With this heterogeneous group composition, we aimed for a comprehensive usability assessment of the presented methods across the clinical field. The recruitment of participants was conducted on a voluntary basis, supported by the research team’s network. It was assumed that the participants had no bias regarding the AAGs, as the methodology and visualization had not been officially released and were therefore not used by the participants at the time of the user test.

### Ethical Considerations

The HCHS study was approved by the Ethics Committee of the Hamburg chamber of physicians (PV5131) and has been registered at ClinicalTrial.gov (NCT03934957).

## Results

### Exemplary Data Analysis

We have generated the AAG for the disease and control group within the HCHS data set based on our definition of a general cardiovascular disease. In this paragraph, we give an exemplary data analysis using the graph and some aspects of the dashboard. This analysis was conducted by the authors of this work independently of the exploration of users during the usability test. The analysis is meant to showcase the usability of the graph representations and is by no means exhaustive. The Neo4j database dumps, configuration files, and user guide can be freely accessed [46]. In addition, the software tool used to generate AAGs was made publicly available [57] and will be presented in an upcoming publication. To assess the compatibility of the presented methods with standard statistical inference, we calculated Pearson correlation coefficients [41], 1-tailed CIs at the confidence level of 95% using the Fisher transformation [45], and *P* values for 1-tailed null hypothesis testing of statistical independence for all associations discussed in the following data analysis. The results can be found in Table S2 in Multimedia Appendix 1.

**Figure 2:**
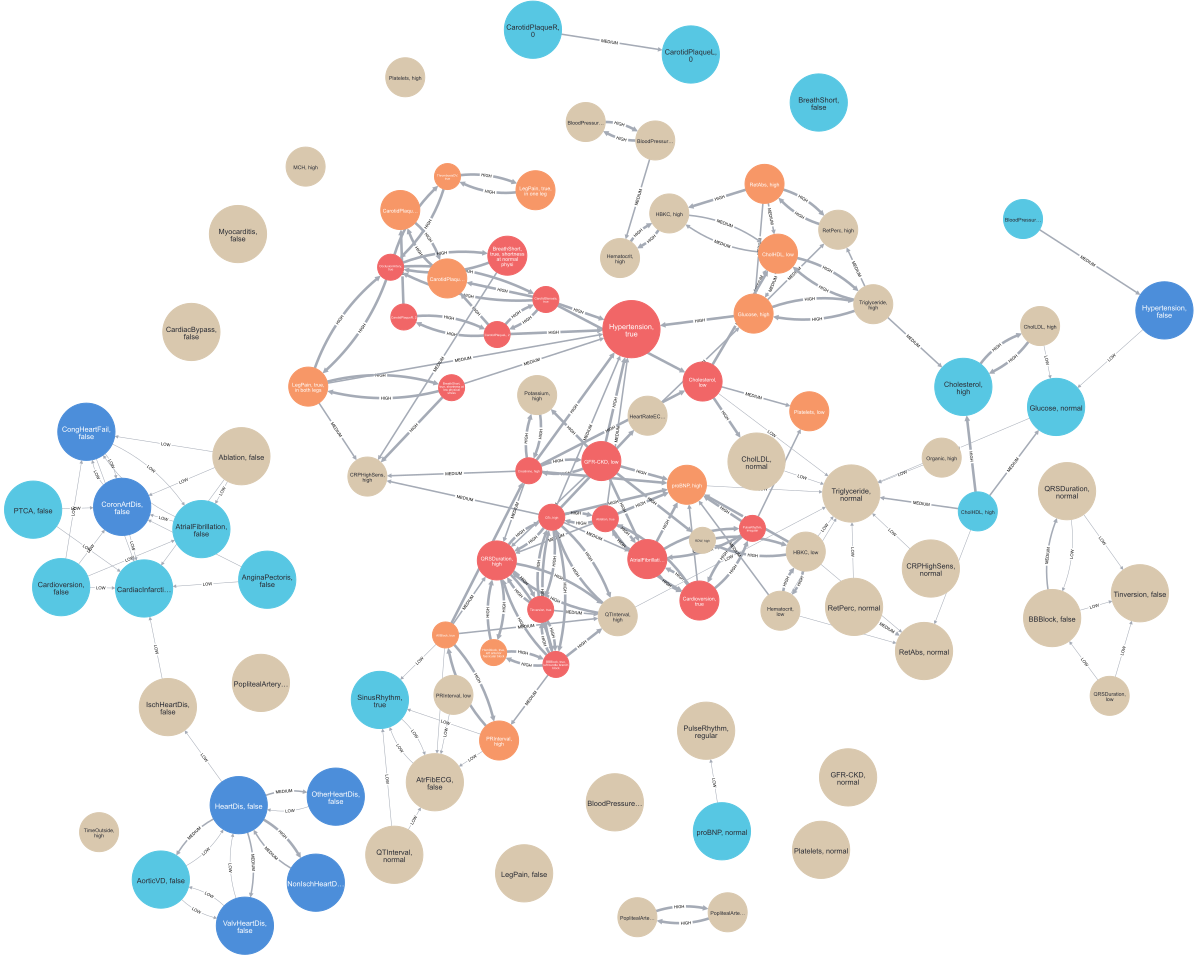
The attribute association graph describing the cardiovascular disease cohort and control group extracted from the Hamburg City Health Study data set. Screenshot taken from the Neo4j Browser. Nodes are depicted as spheres, and edges are depicted as lines between spheres. The color of the sphere indicates the deviation label of the node: vanilla (unrelated), orange (related), red (highly related), turquoise (inverse), and blue (highly inverse). The size of the sphere indicates the frequency label from the smallest (infrequent) to the largest size (highly frequent). The line thickness indicates the type of edge from thinnest (low conditional difference) to thickest (high conditional difference). The text inside the node spheres states the variable name, followed by the value of the attribute. Data and variable descriptions can be found in the ZFDM repository. For a higher-resolution version of this figure, see Multimedia Appendix 3. Variable descriptions are found in Multimedia Appendix 4.

For brevity, we define the cardiovascular disease group as group A and its control group as group B. Group A contains 1917 participants, and group B contains 8083 participants. The generated AAG is shown in Figure 2 [14,46]. The nodes labeled as related or highly related form a cluster in the middle of the graph with the highest density of edges between them. Most of the inverse and highly inverse labeled nodes are primarily located on the periphery of the graph with many interconnections but few connections to the inner cluster. This observation indicates a clear distinction highlighted by the graph between the attributes based on their cooccurrence with cardiovascular disease within the HCHS data set.

**Figure 3:**
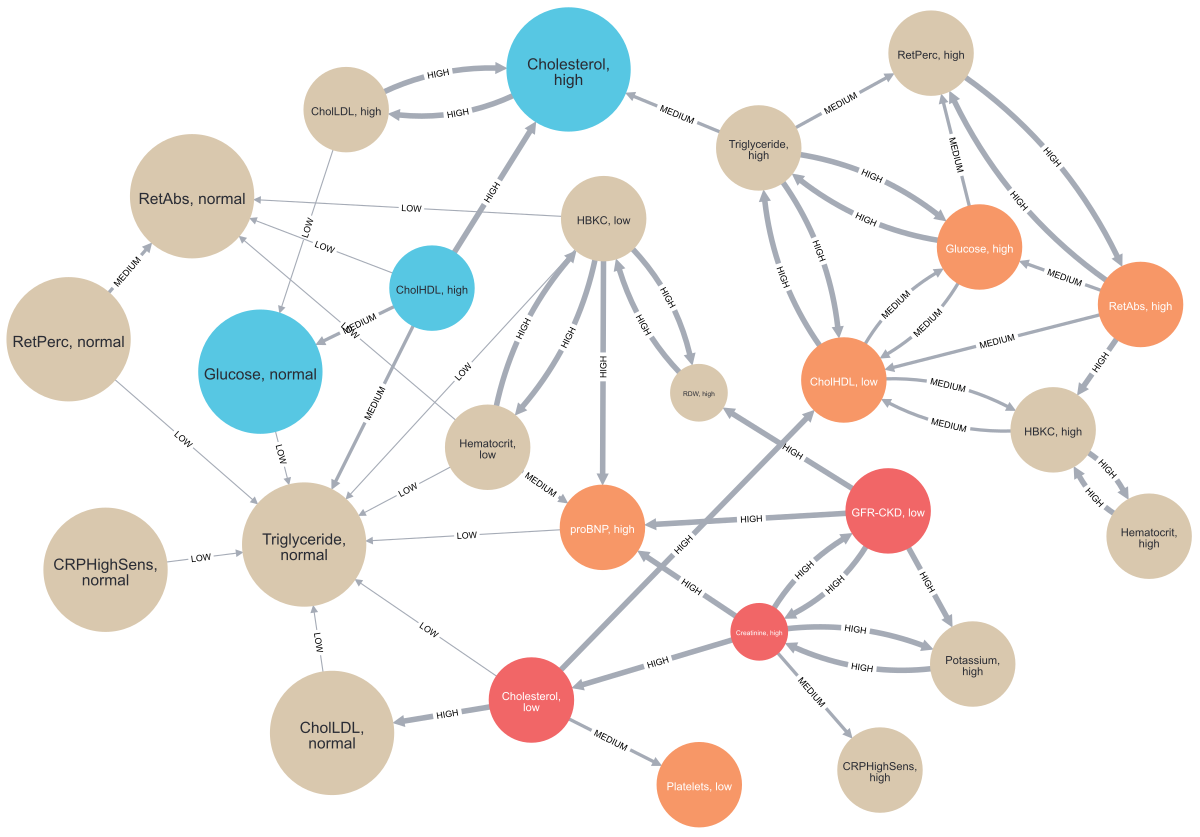
A subgraph of the full attribute association graph describing the cardiovascular disease cohort and control group extracted from the Hamburg City Health Study data set. Screenshot taken from the Neo4j Browser. Only nodes representing laboratory measurements and edges between them are shown. The color of the sphere indicates the deviation label of the node: vanilla (unrelated), orange (related), red (highly related), turquoise (inverse), and blue (highly inverse). The size of the sphere indicates the frequency label from the smallest (infrequent) to the largest size (highly frequent). The line thickness indicates the type of edge from thinnest (low conditional difference) to thickest (high conditional difference). The text inside the node spheres states the variable name, followed by the value of the attribute. Data and variable descriptions can be found in the ZFDM repository. CKD: chronic kidney disease; CRP: C-reactive protein; GFR: glomerular filtration rate; HBKC: hemoglobin level; HDL: high-density lipoprotein; LDL: low-density lipoprotein; proBNP: prohormone of B-type natriuretic peptide. For a higher-resolution version of this figure, see Multimedia Appendix 5. Variable descriptions are found in Multimedia Appendix 4.

For a more detailed analysis of this AAG, we focused on the laboratory results data shown in Figure 3 [14,46]. Within the graph, 3 nodes are labeled as highly related, along with several adjacent nodes labeled as related. The nodes representing glomerular filtration rate <60 mL/min/1.73 m^2^ (“GFR-CKD, low”) and creatinine levels >1.2 mg/dL (“creatine, high”) are identified as highly related and are interconnected. Furthermore, they are also connected to the node representing elevated potassium levels >4.15 mmol (“potassium, high”) through high conditional difference relationships. The presence of a low glomerular filtration rate, high creatinine, and elevated potassium levels are all correlated with chronic kidney disease [58], which in turn is a risk factor for the development of cardiovascular conditions [58,59]. Thus, all 3 laboratory results are associated with heart disease in clinical settings [60], which coincides with the findings presented in this graph. The respective 95% CIs lie fully above 0 for creatine and potassium levels and fully below 0 for the glomerular filtration rate. The relative attribute share of the nodes for glomerular filtration rate <60 mL/min/1.73 m^2^ (“GFR-CKD, low”) in group A is, with 12%, more than twice as high as the relative total share. This indicates missing values for glomerular filtration rate measurements in participant with a cardiovascular condition. The related node in the center of Figure 3 (“proBNP, high”) represents elevated N-terminal prohormone of B-type natriuretic peptide (proBNP) levels >125 ng/L, which were identified as a biomarker for cardiac diseases [61]. With 47%, group A has a 1.7-fold increased relative attribute share for this attribute compared to group B. The associated CI for the Pearson correlation coefficient is strictly positive. The node has 3 incoming edges of high conditional difference. Of these 3 edges, 2 describe the relationship between low glomerular filtration rate and high creatinine levels to elevated proBNP levels. Participants of group B with 1 of these properties are at least 1.6-fold more likely to show elevated proBNP levels >125 ng/L compared to general patients of group B. The same pattern can be observed in group A, which is consistent with the impact of worsening kidney function on proBNP concentration [62,63]. The CIs of the Pearson correlation coefficient of proBNP and glomerular filtration rate is strictly negative, and the CI for creatinine and proBNP levels is fully positive. The third incoming edge is of type high conditional difference. It indicates a relationship between hemoglobin levels <13 g/dL (“HBKC, low”) and elevated proBNP measurements. Although the node for low hemoglobin levels is labeled as unrelated, measurements <13 g/dL appear with a 1.4-fold increase in group B compared to group A. The associated CI is close to, but fully above, 0. Interestingly, participants of both groups with low hemoglobin levels are approximately 1.5-fold more likely to exhibit high proBNP measurements compared to general participants of their group, a phenomenon observed in other studies [64-66]. The Pearson correlation coefficient CI for proBNP and hemoglobin levels are close to, but fully below, 0. Overall, these 3 relationships confirm that while elevated proBNP levels serve as a biomarker for cardiac conditions, other factors may also contribute to its elevation.

**Figure 4:**
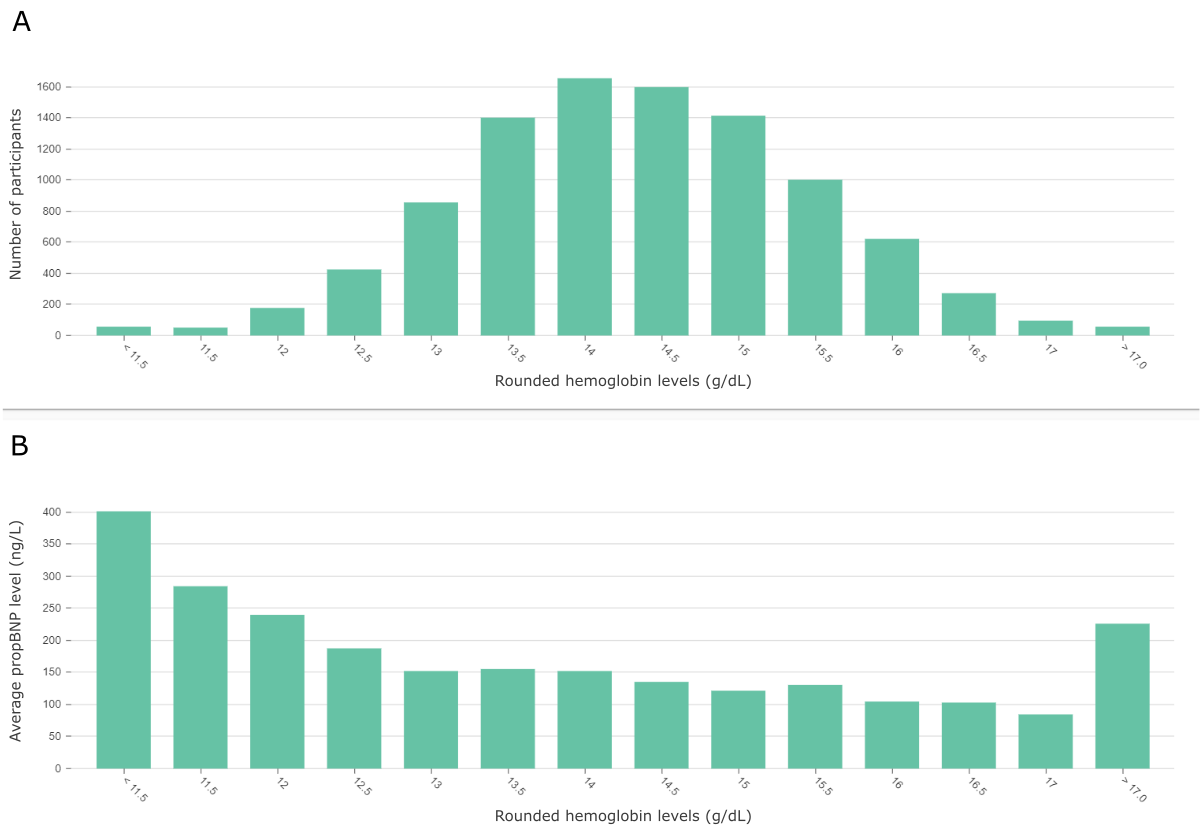
(A) Distribution of hemoglobin levels (g/dL) across all participants of the Hamburg City Health Study data set. (B) The average N-terminal prohormone of B-type natriuretic peptide (proBNP) level (ng/L) per participant of the data set with a rounded hemoglobin level specified on the x-axis. This figure is a screenshot from the dashboard.

Figure 4 was extracted from the dashboard and discloses the relationship of hemoglobin and proBNP levels across the whole data set in more detail. Average proBNP values increase for participants with hemoglobin levels <13 g/dL. Interestingly, proBNP levels also increase in participants with high hemoglobin values >17 g/dL. For further investigation, we returned to the graph and inspected the node (“HBKC, high”) for high hemoglobin levels >15.5 g/dL. This threshold is exceeded by 21.5% of the participants in group A and by only 15.3% of the participants in group B. These observations align with the calculated, strictly positive CI and findings of other studies associating high hemoglobin concentrations with cardiovascular disease [67,68]. The third node (“cholesterol, low”), which is labeled as highly related, can be seen in the lower center of Figure 3. It represents total cholesterol levels <150 mg/dL, which is exhibited by 16.3% of group A and only 5.5% of group B. Conversely, total cholesterol levels >200 mg/dL are observed in 47.3% of group A and 61.2% of group B. As a result, the corresponding node (“cholesterol, high”) is labeled as inversely related.

**Figure 5:**
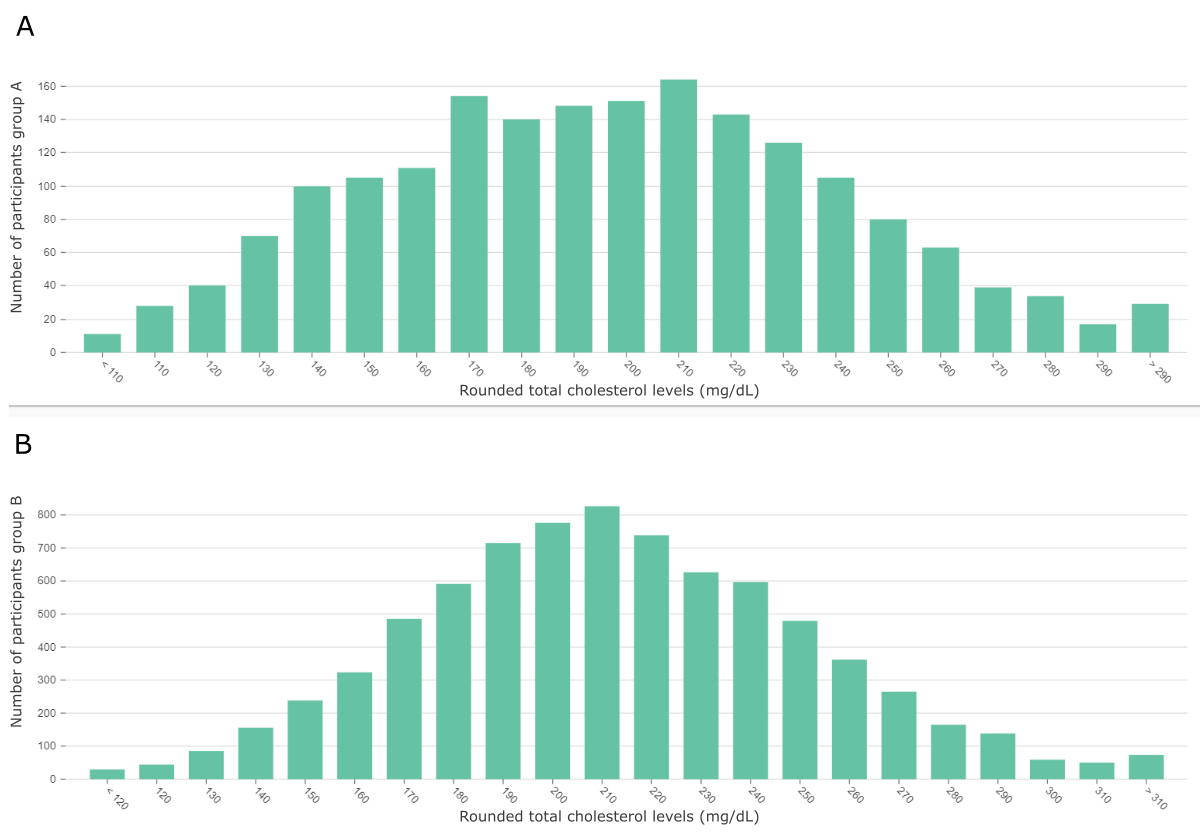
(A) Distribution of total cholesterol levels (mg/dL) for the cardiovascular disease group (group A) and (B) its control group (group B) derived from the Hamburg City Health Study data set. This figure is a screenshot taken from the dashboard.

However, in Figure 5, we can observe that the highest number of participants in both groups exhibit a slightly elevated total cholesterol level of 210 mg/dL. Next, we inspected the 2 nodes (“CholLDL, normal” and “CholLDL, high”) for low-density lipoprotein (LDL) cholesterol levels. Measurements >130 mg/dL (“CholLDL, high”) appear with a 1.3-fold increase in group B. LDL cholesterol levels <130 mg/dL (“CholLDL, normal”) appear in 68.1% of group A and 59.7% of group B. These observations are peculiar because elevated total and LDL cholesterol are commonly recognized as important risk factors for cardiovascular diseases [69-73]. A similar pattern can be inferred from the 2 nodes (“CholHDL, low” and “CholHDL, high”) for measurements of high-density lipoprotein (HDL) cholesterol. Levels <45 mg/dL appear with a 1.7-fold increase in group A, whereas measurements >83 mg/dL showed a 1.8-fold increase in group B. This observation coincides with the widely accepted inverse association of HDL levels with cardiovascular diseases [74,75]. It is noteworthy that the nodes for high LDL and HDL cholesterol levels share an edge with the node for high total cholesterol levels. The same holds true for low HDL, normal LDL, and low total cholesterol measurements. These edges are all labeled with “high conditional difference.” The CIs for all 3 cholesterol measurements and the membership to group A are strictly negative. The CIs for total cholesterol levels and HDL as well as LDL cholesterol measurements are strictly positive, with the correlation coefficient of LDL and total cholesterol being close to 1. In summary, reduced overall cholesterol, LDL cholesterol, and HDL cholesterol levels appear more often in the cardiovascular disease group compared to the control group and are associated with each other. As stated earlier, this observation contradicts the commonly accepted association of elevated overall and LDL cholesterol with cardiovascular diseases. It could be attributed to the widely used therapy with statins [76], which mainly targets the reduction of LDL and overall cholesterol [77]. On the basis of this idea, the high conditional difference relation between elevated creatinine levels and low total cholesterol measurements found in Figure 3 and the associated strictly negative CI for the Pearson correlation coefficient could be explained by statin-associated muscle symptoms [78]. However, additional information about the medication history of the participants would be required and could be a starting point for further investigation.

### User Tests

The participants indicated a work experience in the current field ranging from 1 to 10 years, with an average of 5.8 years. The data exploration tools mostly used by the participants were SPSS (IBM) [79], R [80], and Microsoft Excel (Microsoft) [81]. No users mentioned any prior experience with graph-based statistical analysis tools. The results of the user test can be found in Multimedia Appendix 3.

**Figure 6:**
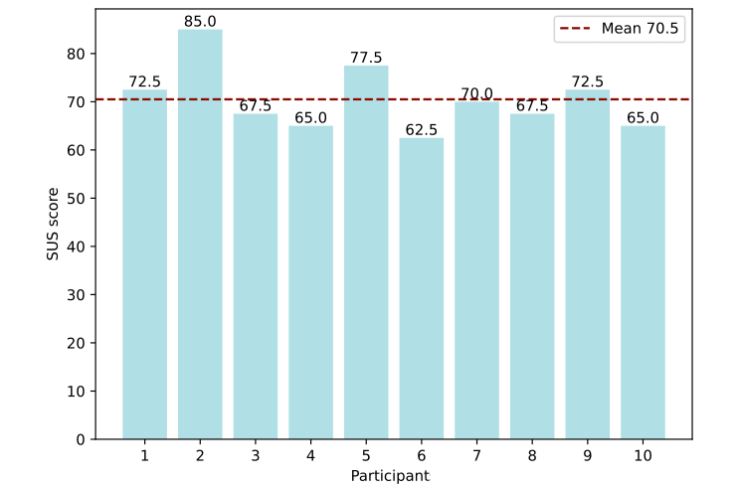
The System Usability Scale (SUS) score for each of the 10 participants of the user test. In addition, the average score is represented by a horizontal dashed line in red.

In Figure 6, the results of the SUS questionnaire are shown and range from 62.5 to 85.0. The mean of 70.5 indicates the passing of usability criteria [82] and a rating of “good” usability [83]. In addition, physicians rated the user-friendliness on a scale from 1 (very bad) to 10 (very good), with a mean of 7.0 in accordance with the SUS results.

**Figure 7:**
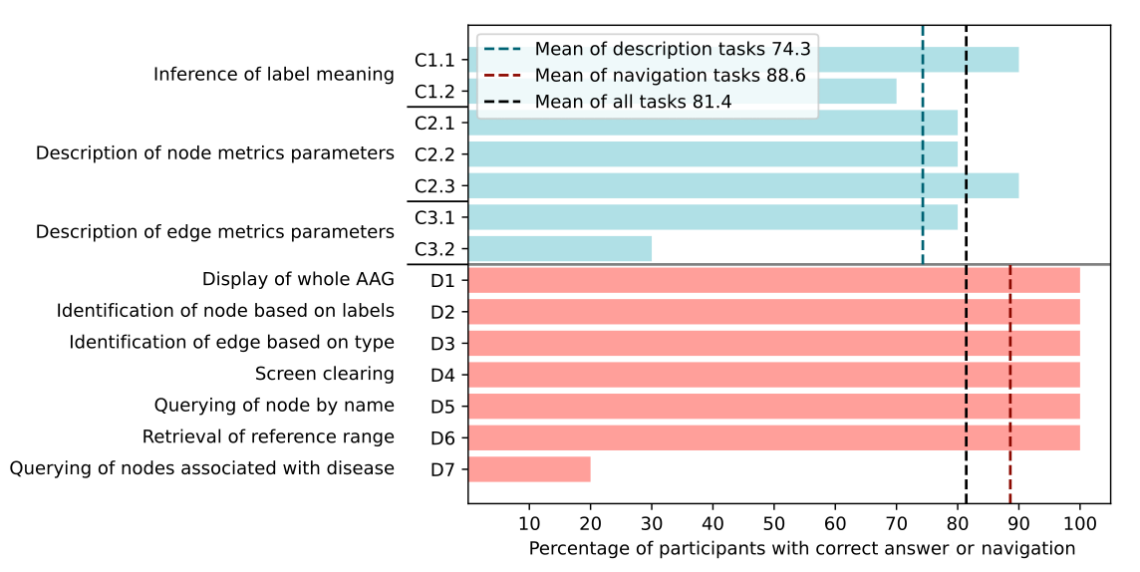
Correct task completion by participants during the user test in percentage. Task numbering is taken from the questionnaire. A short description of the tasks is given on the left. Bars for description and reproduction of labels and metrics (task category 1) are depicted in turquoise. Bars for graph navigation tasks (task category 2) are depicted in pink. Average percentages of correct tasks are plotted as dashed lines for description, navigation, and all tasks.

In Figure 7, the percentage of the 10 participants with successful completion is shown for each user task. The average score across all tasks is 81.4%, with 6 (86%) of 7 navigation tasks being correctly completed by all participants. However, only 20% (2/10) of participants queried successfully for the 10 nodes most statistically associated with the disease group by the quotient of relative attribute shares. Regarding the description tasks of category 1, all but 1 task of reproducing label and parameter meaning was completed by at least 70% (7/10) of users. An exception was task C3.2 where participants should describe the meaning of the edge parameter for the difference of relative conditional share and relative attribute share of the target node. This task was only completed correctly by 30% (3/10) of the participants. In addition, only 30% (3/10) of the participants found the parameter names for nodes understandable, and only 10% (1/10) of the participants classified the edge parameter names as clear.

During the free data exploration, all participants noticed the unusually low levels of total and LDL cholesterol in the cardiovascular disease group compared to the control group, which is also discussed during the exemplary data analysis conducted by the authors. In addition, 40% (4/10) of the participants suspected this association to be caused by medication not represented in the data set. Overall, 60% (6/10) of the participants discussed ECG signals, and 60% (6/10) of the participants discussed kidney metabolism. Moreover, 70% (7/10) of the physicians mentioned the results of their data exploration to be plausible, except for total and LDL cholesterol unprompted. Regarding the answers to the open feedback questions, 80% (8/10) of the participants mentioned the colors and sizes of nodes to be helpful, and 40% (4/10) of the participants referred to the display of attribute connections as edges becoming apparent. Moreover, 30% (3/10) of the participants mentioned the benefit of initial data exploration without the need for numerical values. As to disadvantages of the AAG, 30% (3/10) of the users mentioned the edge definitions being hard to understand, 20% (2/10) assessed the graphs to be too crowded to get a good overview, and 20% (2/10) stated that they would need more practice to use the tool efficiently.

## Discussion

### Principal Findings

In this work, we presented the AAG for visual exploration of medical data sets using disease and control cohorts. The graph structure represents attributes as nodes and identifies as well as visually highlights attributes, which are linked to the observed disease by robust statistical metrics. Relations between attributes are captured as edges by conditional frequencies. As a result, attributes associated with the observed disease are visually clustered and clearly separated from attributes, which are associated with the control group. The graph structure detects and handles missing values without the need for data deletion.

The usability of the AAG and dashboard was assessed using an exemplary data analysis. All but 1 association of laboratory measurements and cardiovascular diseases extracted from the HCHS data set are in line with findings from the literature. The exceptions are unusually low total and LDL cholesterol levels in participants with cardiovascular disease, which might be caused by lipid-lowering therapy. All results extracted from the AAG were confirmed by standard statistical inference using null hypothesis testing and CI for the Pearson correlation coefficient. In addition, a user test with physicians was conducted using the standardized SUS questionnaire, nonstandardized open feedback questions as well as user tasks, and a free data exploration. The SUS score of 70.5% and average successful task completion of 81.4% show a general acceptance and good usability of the AAG. After the initial 30-minute preparation period, all users were able to navigate the graph and could extract medical knowledge that they considered plausible and meaningful. In addition, all participants identified the unusual lipid levels in participants of the cardiovascular disease group and some suspected medication not represented in the data set to be the cause. The encoding of statistical results by color, size, and clustering of nodes as well as thickness of edges was seen as helpful by the users. The users regarded the tool as useful for accessible hypothesis formation during the initial research phase.

### Comparison With Prior Work

Other existing data-driven approaches based on graph structures focus mainly on the connection of different data sources as knowledge graphs [23,24,26] or direct clinical decision support through outcome prediction [27,31,35,38,84-86]. To our knowledge, a graph structure capturing statistical measurements of a medical data set using disease and control cohorts with a clear focus on interpretability and visualization is a novel approach. In addition, as our proposed methods consider single attributes and pairs of attributes, they are robust to high-dimensional data, which pose a problem for many other statistical models applied to the medical domain [87]. We believe that the usability of graph-based visualizations in the medical field is rarely assessed using standardized tests such as the SUS questionnaire. The only other results known to the authors reported a slightly lower SUS score of 64.4 [25].

Regarding the graph-based statistical framework, we see our work closest related to Bayesian networks [29] and association rule learning [36]. While Bayesian networks can hold strong predictive power [88], the choice of prior distribution and sensitivity to data quality can be challenging for clinicians [89]. In association rule learning, conditional relationships between attributes are partially expressed through the confidence parameter, which is quite similar to our methodology in that regard. However, we enrich the added condition with semantics by calculating difference and quotient to the unconditional relative frequencies. Finally, none of the 2 methods measure statistical differences between disease and control cohorts. We believe this to be vital in our approach for generation of insight and adoption in the medical domain.

### Limitations

We intended the AAG and dashboard as a compact visualization for data exploration in the initial phase of research projects. We aimed to incorporate easily interpretable, robust metrics in the form of conditional and unconditional absolute and relative frequencies as well as their deviations between disease and control cohorts. However, because of this choice of metrics, the accuracy could be lower when used in prediction tasks compared to, for example, Bayesian networks or other nonlinear models. In addition, CIs and null hypothesis significance testing play a key role in statistical inference of medical data [90]. They are not incorporated into the methods presented here but could be a follow-up to the initial exploration using the AAG. Finally, temporal data cannot be handled with the proposed methodology in the current form, and Markov models [33] could be applied instead.

Regarding the usability of the visualization, the results of the user test indicate a need for simplification of the parameter names regarding the statistical measurements. In addition, the comparison of conditional and unconditional frequencies captured in the edges of the graph structure was not accessible enough for the users. Moreover, the prompt for retrieval of nodes most associated with one of the groups was considered too lengthy by the users. The authors will incorporate this valuable feedback in the next update iteration of the presented methods.

### Conclusions

In this work, we introduced the AAG, a novel graph-based representation of statistical data combined with a dashboard. These structures can be visually explored and allow for data analysis tailored to the needs of the medical domain. The usability of the graph structure and dashboard was confirmed by user tests conducted with physicians. In addition, the validity of the incorporated statistical analysis was assessed through an exemplary data analysis of a large epidemiological study, and its compatibility with standard statistical methodology and findings from the literature was established. For the future, it might be of interest to enable clinicians in generating their own AAGs without the need for programming experience as an extension to their existing data analysis workflow. To achieve this, we developed a software package [57], which will be presented in an upcoming publication. We think that accessible data analysis and intuitive presentation for clinicians and patients is the way forward in a world of ever-growing data availability and complexity.

## Appendix

Multimedia Appendix 1 Attribute association graph filter criteria, manually adjusted reference ranges, Pearson correlation coefficients, CIs at the confidence level of 95% using the Fisher transformation, and *P* values for 1-tailed null hypothesis testing of statistical independence.

Multimedia Appendix 2 User test questionnaire.

Multimedia Appendix 3 The attribute association graph describing the cardiovascular disease cohort and control group extracted from the Hamburg City Health Study data set. Screenshot taken from the Neo4j Browser. Nodes are depicted as spheres, and edges are depicted as lines between spheres. The color of the sphere indicates the deviation label of the node: vanilla (unrelated), orange (related), red (highly related), turquoise (inverse), and blue (highly inverse). The size of the sphere indicates the frequency label from the smallest (infrequent) to the largest size (highly frequent). The line thickness indicates the type of edge from thinnest (low conditional difference) to thickest (high conditional difference). The text inside the node spheres states the variable name, followed by the value of the attribute. Data and variable descriptions can be found in the ZFDM repository.

Multimedia Appendix 4 Variable descriptions for Figures 2 and 3.

Multimedia Appendix 5 A subgraph of the full attribute association graph describing the cardiovascular disease cohort and control group extracted from the Hamburg City Health Study data set. Screenshot taken from the Neo4j Browser. Only nodes representing laboratory measurements and edges between them are shown. The color of the sphere indicates the deviation label of the node: vanilla (unrelated), orange (related), red (highly related), turquoise (inverse), and blue (highly inverse). The size of the sphere indicates the frequency label from the smallest (infrequent) to the largest size (highly frequent). The line thickness indicates the type of edge from thinnest (low conditional difference) to thickest (high conditional difference). The text inside the node spheres states the variable name, followed by the value of the attribute. Data and variable descriptions can be found in the ZFDM repository. CKD: chronic kidney disease; CRP: C-reactive protein; GFR: glomerular filtration rate; HBKC: hemoglobin level; HDL: high-density lipoprotein; LDL: low-density lipoprotein; proBNP: prohormone of B-type natriuretic peptide.

Multimedia Appendix 6 Results of the user test.

## Abbreviations

AAG: attribute association graph
CRP: C-reactive protein
ECG: electrocardiography
HCHS: Hamburg City Health Study
HDL: high-density lipoprotein
LDL: low-density lipoprotein
proBNP: prohormone of B-type natriuretic peptide
SUS: System Usability Scale

## Data Availability

The data sets generated and analyzed during this study are available in the ZFDM repository [46]. The HCHS data set itself is not publicly available due participant data privacy. The attribute association graph (AAG) and dashboard data as Neo4j dumps, Neo4j Bloom configuration as JSON files, as well as a detailed installation and user guide as PDF file and descriptions for all variables of the Hamburg City Health Study data subset can be found in the repository [46]. Adjusted reference ranges and filter criteria for the AAGs, Pearson correlation coefficients, as well as the user test questionnaire and results can be found in Multimedia Appendices 1, 2 and 6. Code repository, Python package, and software tool to create custom AAGs [57] will be described in an upcoming publication.

## References

[1] Martin-Sanchez F, Verspoor K (2014). Big data in medicine is driving big changes. Yearb Med Inform.

[2] Mallappallil M, Sabu J, Gruessner A, Salifu M (2020). A review of big data and medical research. SAGE Open Med.

[3] Egger J, Gsaxner C, Pepe A, Pomykala KL, Jonske F, Kurz M, Li J, Kleesiek J (2022). Medical deep learning-a systematic meta-review. Comput Methods Programs Biomed.

[4] Baldi P (2018). Deep learning in biomedical data science. Annu Rev Biomed Data Sci.

[5] Shen D, Wu G, Suk HI (2017). Deep learning in medical image analysis. Annu Rev Biomed Eng.

[6] Price WN (2018). Big data and black-box medical algorithms. Sci Transl Med.

[7] Poon AI, Sung JJ (2021). Opening the black box of AI-medicine. J Gastroenterol Hepatol.

[8] Rudin C (2019). Stop explaining black box machine learning models for high stakes decisions and use interpretable models instead. Nat Mach Intell.

[9] Amann J, Blasimme A, Vayena E, Frey D, Madai VI, Precise4Q consortium (2020). Explainability for artificial intelligence in healthcare: a multidisciplinary perspective. BMC Med Inform Decis Mak.

[10] Wanderer JP, Nelson SE, Ehrenfeld JM, Monahan S, Park S (2016). Clinical data visualization: the current state and future needs. J Med Syst.

[11] Badgeley MA, Shameer K, Glicksberg BS, Tomlinson MS, Levin MA, McCormick PJ, Kasarskis A, Reich DL, Dudley JT (2016). EHDViz: clinical dashboard development using open-source technologies. BMJ Open.

[12] Torsvik T, Lillebo B, Mikkelsen G (2013). Presentation of clinical laboratory results: an experimental comparison of four visualization techniques. J Am Med Inform Assoc.

[13] Schrodt J, Dudchenko A, Knaup-Gregori P, Ganzinger M (2020). Graph-representation of patient data: a systematic literature review. J Med Syst.

[14] GenAI apps, grounded in your data. Neo4j Graph Data Platform: The Leader in Graph Databases.

[15] Almabdy S (2018). Comparative analysis of relational and graph databases for social networks. Proceedings of the 1st International Conference on Computer Applications & Information Security.

[16] Khan W, ahmed E, Shahzad W (2017). Predictive performance comparison analysis of relational and NoSQL graph databases. Int J Adv Comput Sci Appl.

[17] NeoDash - dashboard builder for Neo4j. Neo4j.

[18] GraphXR: visual analytics, graph BI, and more. Kineviz.

[19] Neo4j bloom. Neo4j Graph Data Platform.

[20] Home page. Graphviz.

[21] Rajabi E, Kafaie S (2022). Knowledge graphs and explainable AI in healthcare. Information.

[22] Fabregat A, Korninger F, Viteri G, Sidiropoulos K, Marin-Garcia P, Ping P, Wu G, Stein L, D'Eustachio P, Hermjakob H (2018). Reactome graph database: efficient access to complex pathway data. PLoS Comput Biol.

[23] Gütebier L, Bleimehl T, Henkel R, Munro J, Müller S, Morgner A, Laenge J, Pachauer A, Erdl A, Weimar J, Walther Langendorf K, Vialard V, Liebig T, Preusse M, Waltemath D, Jarasch A (2022). CovidGraph: a graph to fight COVID-19. Bioinformatics.

[24] Dedié A, Bleimehl T, Täger J, Preusse M, de Angelis MH, Jarasch A (2021). DZDconnect: mit vernetzten daten gegen diabetes. Diabetologe.

[25] He X, Zhang R, Rizvi R, Vasilakes J, Yang X, Guo Y, He Z, Prosperi M, Huo J, Alpert J, Bian J (2019). ALOHA: developing an interactive graph-based visualization for dietary supplement knowledge graph through user-centered design. BMC Med Inform Decis Mak.

[26] Lysenko A, Roznovăţ IA, Saqi M, Mazein A, Rawlings CJ, Auffray C (2016). Representing and querying disease networks using graph databases. BioData Min.

[27] McLachlan S, Dube K, Hitman GA, Fenton NE, Kyrimi E (2020). Bayesian networks in healthcare: distribution by medical condition. Artif Intell Med.

[28] Burnside E, Rubin D, Shachter R (2000). A Bayesian network for mammography. Proc AMIA Symp.

[29] Kitson NK, Constantinou AC, Guo Z, Liu Y, Chobtham K (2023). A survey of Bayesian network structure learning. Artif Intell Rev.

[30] Smith VA, Yu J, Smulders TV, Hartemink AJ, Jarvis ED (2006). Computational inference of neural information flow networks. PLoS Comput Biol.

[31] Burnside ES (2005). Bayesian networks: computer-assisted diagnosis support in radiology. Acad Radiol.

[32] Ke X, Keenan K, Smith VA (2022). Treatment of missing data in Bayesian network structure learning: an application to linked biomedical and social survey data. BMC Med Res Methodol.

[33] Sonnenberg FA, Beck JR (1993). Markov models in medical decision making: a practical guide. Med Decis Making.

[34] Beck JR, Pauker SG (1983). The Markov process in medical prognosis. Med Decis Making.

[35] Hogendoorn W, Moll FL, Sumpio BE, Hunink MG (2016). Clinical decision analysis and Markov modeling for surgeons: an introductory overview. Ann Surg.

[36] Agrawal R, Imieliński T, Swami A (1993). Mining association rules between sets of items in large databases. SIGMOD Rec.

[37] Stilou S, Bamidis PD, Maglaveras N, Pappas C (2001). Mining association rules from clinical databases: an intelligent diagnostic process in healthcare. Stud Health Technol Inform.

[38] Harahap M, Husein AM, Aisyah S, Lubis FR, Wijaya BA (2018). Mining association rule based on the diseases population for recommendation of medicine need. J Phys Conf Ser.

[39] Johnston BC, Alonso-Coello P, Friedrich JO, Mustafa RA, Tikkinen KA, Neumann I, Vandvik PO, Akl EA, da Costa BR, Adhikari NK, Dalmau GM, Kosunen E, Mustonen J, Crawford MW, Thabane L, Guyatt GH (2016). Do clinicians understand the size of treatment effects? a randomized survey across 8 countries. CMAJ.

[40] Pearson K (2009). X. On the criterion that a given system of deviations from the probable in the case of a correlated system of variables is such that it can be reasonably supposed to have arisen from random sampling. Lond Edinb Dubl Phil Mag J.

[41] Pearson K (1997). Note on regression and inheritance in the case of two parents. Proc R Soc Lond.

[42] Emmanuel T, Maupong T, Mpoeleng D, Semong T, Mphago B, Tabona O (2021). A survey on missing data in machine learning. J Big Data.

[43] Wood AM, White IR, Thompson SG (2004). Are missing outcome data adequately handled? a review of published randomized controlled trials in major medical journals. Clin Trials.

[44] Padilla O, Abadie J Normal laboratory values. MSD Manual Professional Version.

[45] Fisher RA (1915). Frequency distribution of the values of the correlation coefficient in samples from an indefinitely large population. Biometrika.

[46] Bellmann L, Wiederhold AJ, Trübe L, Twerenbold R, Ückert F, Gottfried K Introducing attribute association graphs to facilitate medical data exploration: development and evaluation using epidemiological study data. Universität Hamburg.

[47] Jagodzinski A, Johansen C, Koch-Gromus U, Aarabi G, Adam G, Anders S, Augustin M, der Kellen RB, Beikler T, Behrendt C, Betz CS, Bokemeyer C, Borof K, Briken P, Busch C, Büchel C, Brassen S, Debus ES, Eggers L, Fiehler J, Gallinat J, Gellißen S, Gerloff C, Girdauskas E, Gosau M, Graefen M, Härter M, Harth V, Heidemann C, Heydecke G, Huber TB, Hussein Y, Kampf MO, von dem Knesebeck O, Konnopka A, König HH, Kromer R, Kubisch C, Kühn S, Loges S, Löwe B, Lund G, Meyer C, Nagel L, Nienhaus A, Pantel K, Petersen E, Püschel K, Reichenspurner H, Sauter G, Scherer M, Scherschel K, Schiffner U, Schnabel RB, Schulz H, Smeets R, Sokalskis V, Spitzer MS, Terschüren C, Thederan I, Thoma T, Thomalla G, Waschki B, Wegscheider K, Wenzel J, Wiese S, Zyriax B, Zeller T, Blankenberg S (2020). Rationale and design of the Hamburg city health study. Eur J Epidemiol.

[48] HCHS study record. ClinicalTrials.

[49] Home page. Systematized Nomenclature of Medicine (SNOMED).

[50] Huff SM, Rocha RA, McDonald CJ, de Moor GJ, Fiers T, Bidgood WD Jr, Forrey AW, Francis WG, Tracy WR, Leavelle D, Stalling F, Griffin B, Maloney P, Leland D, Charles L, Hutchins K, Baenziger J (1998). Development of the Logical Observation Identifier Names and Codes (LOINC) vocabulary. J Am Med Inform Assoc.

[51] Helminski D, Kurlander JE, Renji AD, Sussman JB, Pfeiffer PN, Conte ML, Gadabu OJ, Kokaly AN, Goldberg R, Ranusch A, Damschroder LJ, Landis-Lewis Z (2022). Dashboards in health care settings: protocol for a scoping review. JMIR Res Protoc.

[52] Wu DT, Vennemeyer S, Brown K, Revalee J, Murdock P, Salomone S, France A, Clarke-Myers K, Hanke SP (2019). Usability testing of an interactive dashboard for surgical quality improvement in a large congenital heart center. Appl Clin Inform.

[53] Elm JJ, Daeschler M, Bataille L, Schneider R, Amara A, Espay AJ, Afek M, Admati C, Teklehaimanot A, Simuni T (2019). Feasibility and utility of a clinician dashboard from wearable and mobile application Parkinson's disease data. NPJ Digit Med.

[54] Brooke J, Jordan PW, Thomas B, McClelland IL, Weerdmeester B (1996). SUS: a 'quick and dirty' usability scale. Usability Evaluation In Industry.

[55] Coe AM, Ueng W, Vargas JM, David R, Vanegas A, Infante K, Trivedi M, Yi H, Dimond J, Crew KD, Kukafka R (2016). Usability testing of a web-based decision aid for breast cancer risk assessment among multi-ethnic women. AMIA Annu Symp Proc.

[56] Hirschmann J, Sedlmayr B, Zierk J, Rauh M, Metzler M, Prokosch HU, Toddenroth D (2017). Evaluation of an interactive visualization tool for the interpretation of pediatric laboratory test results. Stud Health Technol Inform.

[57] Bellmann L GraphXplore code repository. GitHub.

[58] Metra M, Cotter G, Gheorghiade M, Dei Cas L, Voors AA (2012). The role of the kidney in heart failure. Eur Heart J.

[59] Vindhyal MR, Khayyat S, Shaaban A, Duran BA, Kallail KJ (2018). Decreased renal function is associated with heart failure readmissions. Cureus.

[60] Wannamethee SG, Shaper AG, Perry IJ (1997). Serum creatinine concentration and risk of cardiovascular disease: a possible marker for increased risk of stroke. Stroke.

[61] Panagopoulou V, Deftereos S, Kossyvakis C, Raisakis K, Giannopoulos G, Bouras G, Pyrgakis V, Cleman MW (2013). NTproBNP: an important biomarker in cardiac diseases. Curr Top Med Chem.

[62] Srisawasdi P, Vanavanan S, Charoenpanichkit C, Kroll MH (2010). The effect of renal dysfunction on BNP, NT-proBNP, and their ratio. Am J Clin Pathol.

[63] Takase H, Dohi Y (2014). Kidney function crucially affects B-type natriuretic peptide (BNP), N-terminal proBNP and their relationship. Eur J Clin Invest.

[64] Willis MS, Lee ES, Grenache DG (2005). Effect of anemia on plasma concentrations of NT-proBNP. Clin Chim Acta.

[65] Karakoyun I, Colak A, Arslan FD, Hasturk AG, Duman C (2017). Anemia considerations when assessing natriuretic peptide levels in ED patients. Am J Emerg Med.

[66] Hogenhuis J, Voors AA, Jaarsma T, Hoes AW, Hillege HL, Kragten JA, van Veldhuisen DJ (2007). Anaemia and renal dysfunction are independently associated with BNP and NT-proBNP levels in patients with heart failure. Eur J Heart Fail.

[67] Chonchol M, Nielson C (2008). Hemoglobin levels and coronary artery disease. Am Heart J.

[68] Lee G, Choi S, Kim K, Yun J, Son JS, Jeong S, Kim SM, Park SM (2018). Association of hemoglobin concentration and its change with cardiovascular and all-cause mortality. J Am Heart Assoc.

[69] Verschuren WM, Jacobs DR, Bloemberg BP, Kromhout D, Menotti A, Aravanis C, Blackburn H, Buzina R, Dontas AS, Fidanza F (1995). Serum total cholesterol and long-term coronary heart disease mortality in different cultures. Twenty-five-year follow-up of the seven countries study. JAMA.

[70] Anderson KM, Castelli WP, Levy D (1987). Cholesterol and mortality. 30 years of follow-up from the Framingham study. JAMA.

[71] Houterman S, Verschuren WM, Hofman A, Witteman JC (1999). Serum cholesterol is a risk factor for myocardial infarction in elderly men and women: the Rotterdam study. J Intern Med.

[72] Ference BA, Ginsberg HN, Graham I, Ray KK, Packard CJ, Bruckert E, Hegele RA, Krauss RM, Raal FJ, Schunkert H, Watts GF, Borén J, Fazio S, Horton JD, Masana L, Nicholls SJ, Nordestgaard BG, van de Sluis B, Taskinen M, Tokgözoglu L, Landmesser U, Laufs U, Wiklund O, Stock JK, Chapman MJ, Catapano AL (2017). Low-density lipoproteins cause atherosclerotic cardiovascular disease. 1. Evidence from genetic, epidemiologic, and clinical studies. A consensus statement from the European Atherosclerosis Society Consensus Panel. Eur Heart J.

[73] Abdullah SM, Defina LF, Leonard D, Barlow CE, Radford NB, Willis BL, Rohatgi A, McGuire DK, de Lemos JA, Grundy SM, Berry JD, Khera A (2018). Long-term association of low-density lipoprotein cholesterol with cardiovascular mortality in individuals at low 10-year risk of atherosclerotic cardiovascular disease. Circulation.

[74] Gordon T, Castelli WP, Hjortland MC, Kannel WB, Dawber TR (1977). High density lipoprotein as a protective factor against coronary heart disease. The Framingham study. Am J Med.

[75] Di Angelantonio E, Sarwar N, Perry P, Kaptoge S, Ray KK, Thompson A, Wood AM, Lewington S, Sattar N, Packard CJ, Collins R, Thompson SG, Danesh J, Emerging Risk Factors Collaboration (2009). Major lipids, apolipoproteins, and risk of vascular disease. JAMA.

[76] Gu Q, Paulose-Ram R, Burt VL, Kit BK (2014). Prescription cholesterol-lowering medication use in adults aged 40 and over: United States, 2003-2012. NCHS Data Brief.

[77] Jafri H, Alsheikh-Ali AA, Karas RH (2010). Meta-analysis: statin therapy does not alter the association between low levels of high-density lipoprotein cholesterol and increased cardiovascular risk. Ann Intern Med.

[78] Ward NC, Watts GF, Eckel RH (2019). Statin toxicity. Circ Res.

[79] SPSS software. IBM Corp.

[80] R Core Team The R project for statistical computing. R Foundation.

[81] Microsoft Excel spreadsheet software. Microsoft 365.

[82] Bangor A, Kortum PT, Miller JT (2008). An empirical evaluation of the system usability scale. Int J Hum Comput Interact.

[83] Bangor A, Kortum P, Miller J (2009). Determining what individual SUS scores mean: adding an adjective rating scale. J Use Exp.

[84] Liu C, Wang F, Hu J, Xiong H (2015). Temporal phenotyping from longitudinal electronic health records: a graph based framework. Proceedings of the 21th ACM SIGKDD International Conference on Knowledge Discovery and Data Mining.

[85] Zhang S, Liu L, Li H, Xiao Z, Cui L (2016). MTPGraph: a data-driven approach to predict medical risk based on temporal profile graph. Proceedings of the 2016 IEEE International Conference on Trust, Security and Privacy in Computing and Communications.

[86] Rotmensch M, Halpern Y, Tlimat A, Horng S, Sontag D (2017). Learning a health knowledge graph from electronic medical records. Sci Rep.

[87] Berisha V, Krantsevich C, Hahn PR, Hahn S, Dasarathy G, Turaga P, Liss J (2021). Digital medicine and the curse of dimensionality. NPJ Digit Med.

[88] Park E, Chang H, Nam HS (2018). A Bayesian network model for predicting post-stroke outcomes with available risk factors. Front Neurol.

[89] Kyrimi E, Dube K, Fenton N, Fahmi A, Neves MR, Marsh W, McLachlan S (2021). Bayesian networks in healthcare: what is preventing their adoption?. Artif Intell Med.

[90] Ioannidis JP (2019). What have we (not) learnt from millions of scientific papers with *P* values?. Am Stat.

