# Supplementary material for: Introducing Attribute Association Graphs to Facilitate Medical Data Exploration: Development and Evaluation Using Epidemiological Study Data
Source: JMIR Med Inform. 2024 Jul 24;12:e49865. doi: 10.2196/49865 (PMC11306949; doi:10.2196/49865)
Supplement: Multimedia Appendix 1 [file medinform_v12i1e49865_app1.docx]

# Introducing Attribute Association Graphs to Facilitate Medical Data Exploration: Development and Evaluation Using Epidemiological Study Data

**Multimedia Appendix 1**

## **Attribute association graph filter criteria**

The nodes and edges of the attribute association graph are filtered to highlight the most relevant attributes and relationships thereof. For the filtering of nodes, we impose a minimum on the relative total share of 1% in all groups. Nodes with a relative total share of less than 5% in all groups must show a difference of relative attribute shares of at least 10% or a quotient of at least 1.5. All remaining nodes must have a difference of at least 5% or a quotient of at least 1.2. Regarding the filtering of edges, we distinguish between four cases: Edges with a relative conditional share below 30% for all groups must have a quotient of relative conditional share and relative attribute share of the target of at least 2.0 for all participant groups. Edges with a relative conditional share of at least 95% for some group, must exhibit a difference of at least 2% for at least one group. Edges with a relative conditional share of at least 80% for all groups, must have a difference of at least 1% for all groups. All edges with a relative conditional share of at least 30% for some participant group, must exhibit a difference of at least 10% for all groups or a quotient of at least 1.5 for all groups.

## **Adjusted reference ranges**

**Table S1.** Variables for which the calculated reference ranges were adjusted. Originally calculated (middle) and adjusted ranges (right) are given. Adjustments were taken from the MSD Manual [44].

| Variable | Calculated range | Adjusted range |
| --- | --- | --- |
| Cholesterol | [167.35;250.56] | [150;199] |
| CholLDL | [84.26;158.36] | [0;130] |
| Creatinine | [0.64;1.09] | [0.5;1.2] |
| CRP | [17.19;58.05] | [0;8] |
| CRPHighSens | [-0.2;0.69] | [0;0.2] |
| fT4 | [12.64;21.3] | [9;24] |
| GFR-CKD | [68.61;97.5] | [60;120] |
| Glucose | [75.3;117.08] | [70;105] |
| Leukocytes | [4.36;8.14] | [0;20] |
| MCH | [28.62;31.9] | [28;32] |
| MCHC | [32.52;34.39] | [32;36] |
| proBNP | [-117.24;405.32] | [0;125] |
| RDW | [12.6;14.46] | [12;15] |
| RetAbs | [45.21;84.29] | [23;90] |
| RetIdx | [0.91;1.7] | [0.5;2.5] |
| RetPerc | [0.97;1.77] | [0.5;1.5] |
| TimeOutside | [1.04;5.24] | [2;4] |
| Triglyceride | [47.2;188.81] | [0;175] |
| TSH | [0.28;2.43] | [0.5;5] |

## **Correlation Coefficients**

We aimed to compare our approach of relative shares deviating between disease and control cohort with standard statistical inference. For this purpose, we calculated the Pearson correlation coefficient [41] for all associations described in the manuscript. We added confidence intervals at confidence level of 0.95 using the Fisher transformation [45], and *P* values for 1-tailed null hypothesis testing of statistical independence. Patients with missing data for at least one of the described variables are removed from the analysis. The first eight associations are calculated between a metric variable of the HCHS dataset and the added Boolean for membership to the cardiovascular disease cohort. The last six associations are calculated between two variables of the dataset.

**Table S2**. Pearson correlation coefficient of two variables, type of null hypothesis, P value of null hypothesis, and confidence interval at confidence level 0.95. CVD describes variable for membership to cardiovascular disease cohort.

| First variable | Second variable | Pearson correlation coefficient | 1-tailed null hypothesis type | *P* value | Confidence interval at 95% |
| --- | --- | --- | --- | --- | --- |
| CVD | GFR-CKD | -0.183 | ρ ≥ 0 | 1.29e-32 | [-1.0;-0.158] |
| CVD | Creatinine | 0.149 | ρ ≤ 0 | 4.51e-22 | [0.123;1.0] |
| CVD | Potassium | 0.102 | ρ ≤ 0 | 2.62e-11 | [0.077;1.0] |
| CVD | proBNP | 0.271 | ρ ≤ 0 | 8.48e-71 | [0.247;1.0] |
| CVD | Cholesterol | -0.171 | ρ ≥ 0 | 1.02e-28 | [-1.0;-0.146] |
| CVD | CholHDL | -0.127 | ρ ≥ 0 | 1.66e-16 | [-1.0;-0.101] |
| CVD | CholLDL | -0.151 | ρ ≥ 0 | 8.25e-23 | [-1.0;-0.126] |
| CVD | HBKC | 0.089 | ρ ≤ 0 | 4.94e-9 | [0.064;1.0] |
| proBNP | GFR-CKD | -0.278 | ρ ≥ 0 | 3.80e-74 | [-1.0;-0.254] |
| proBNP | Creatinine | 0.248 | ρ ≤ 0 | 5.24e-59 | [0.224;1.0] |
| proBNP | HBKC | -0.105 | ρ ≥ 0 | 6.86e-12 | [-1.0;-0.080] |
| Cholesterol | CholLDL | 0.926 | ρ ≤ 0 | < 1.00e-80 | [0.923;1.0] |
| Cholesterol | CholHDL | 0.285 | ρ ≤ 0 | 3.00e-78 | [0.261;1.0] |
| Cholesterol | Creatinine | -0.158 | ρ ≥ 0 | 9.62e-25 | [-1.0;-0.133] |
