# Supplementary material for: Introducing Attribute Association Graphs to Facilitate Medical Data Exploration: Development and Evaluation Using Epidemiological Study Data
Source: JMIR Med Inform. 2024 Jul 24;12:e49865. doi: 10.2196/49865 (PMC11306949; doi:10.2196/49865)
Supplement: Multimedia Appendix 2 [file medinform_v12i1e49865_app2.pdf]

# Semi-Structured Interview for GraphXplore Usability

## User Testing

### Interview - Introduction (A)

1. Basic introduction.

Thank you for taking the time to participate in the following survey. We would like to present you GraphXplore, a form of data visualization based on graphs over the next 30 minutes. We aim to test the usability of the visualizations with clinical/scientific users using the Hamburg City Health Study (HCHS) as an example.

First, we will ask you questions about your experience with data visualizations in general, followed by a brief introduction of GraphXplore and the Neo4j or Bloom application for visualization. Afterwards, we invite you to use this information to complete three tasks. This way, you will familiarize yourself with GraphXplore before answering a questionnaire (System Usability Scale (SUS)) with 10 questions.

Finally, we would like to ask you five interview questions. We will record your answers in an anonymized form and use them later for the overall evaluation of GraphXplore. Your participation is voluntary and can be terminated at any time. If you cannot or do not want to answer a question, please let us know at any time.

By participating, you help us assess the value of the visualization tool and improve its user-friendliness.

1. Do you have any questions about the outlined procedure?

| |
|---|
| ☐ Yes |
| ☐ No |

2. Do you agree to the data survey and the recording of the answers?

| |
|---|
| ☐ Yes |
| ☐ No |

## Interview - General Questions (B)

1. Have you used Neo4j or Neo4j Bloom before?

| |
|---|
| ☐ Yes |
| ☐ No |

2. How long do you work in the current field/area (or what is your level of experience in your working field)?

| |
|---|
| |

3. In which of the named areas are you regularly active? (multi-selection is possible)

| |
|---|
| ☐ Clinic |
| ☐ Science/Research |
| ☐ None of the above |

4. Have you used data exploration tools before?

| |
|---|
| ☐ Yes |
| ☐ No |

5. If B4 = Yes: How often do you use data exploration tools?

| |
|---|
| ☐ Daily or multiple times a week |
| ☐ At least once a month |
| ☐ A few times a year |
| ☐ I used to use them frequently, but not anymore |

6. If B4 = Yes: List some data exploration tools, you have used or currently use.

| |
|---|
| |

## GraphXplore/Neo4j Bloom Introduction and Tasks

Take 30 minutes to read the following topics:

1. Read the overview introduction to get a basic understanding of the Neo4j Bloom application
2. Read the GraphXplore user guide chapter “Attribute association graphs” (p. 5-12).
3. Do you have any questions?

| |
|---|
| ☐ Yes |
| ☐ No |

### Task 1: User Manual (C)

C1. Let's look at the illustration on page 8 of the guide.

1. Which node appears very frequently and is notably prevalent in the healthy group?

| |
|---|
| |

2. What parameter configuration do you expect for such a node?

| |
|---|
| |

C2. Answer the following questions:

1. Describe the parameter 'groupRelShareTotals' in your own words?

| |
|---|
| |

2. Describe the parameter 'groupRelShareAttrs' in your own words?

| |
|---|
| |

3. Describe the parameter 'groupAbsCounts' in your own words?

| |
|---|
| |

4. Are the names of these parameters clear and understandable?

| |
|---|
| |

C3. Now, answer the following questions about relationships:

1. Describe the parameter 'groupRelShareConds' in your own words?

| |
|---|
| |

2. Describe the parameter 'groupDiffTargets' in your own words?

| |
|---|
| |

3. Are the names of these parameters clear and understandable for you?

| |
|---|
| |

### Task 2: Node Identification by Frequency (D)

1. Please show the whole graph on the screen by using the search bar.

| |
|---|
| ☐ Done without help |
| ☐ Done with help |

2. Identify the name and value of the node which is labeled as highly frequent and highly related.

| |
|---|
| ☐ correct |
| ☐ not correct |

3. Find a relationship which is labeled as high conditional difference (“high_cond_diff“) and name its source and target node.

| |
|---|
| ☐ correct |
| ☐ not correct |

4. Please clear the scene.

| |
|---|
| ☐ Done without help |
| ☐ Done with help |

5. Search for node with the name “CRPHighSens”.

| |
|---|
| ☐ Done without help |
| ☐ Done with help |

6. Above which threshold are measurements of this variable considered as “high”?

| |
|---|
| ☐ correct |
| ☐ not correct |

7. Please clear the scene. Show the top 10 attributes for the disease group and their relationships.

| |
|---|
| ☐ Done without help |
| ☐ Done with help |

### Task 3: Exploratory Data Analysis (E)

1. Please explore the graph (top 10 attributes) using your medical knowledge and verbalize your thoughts as you do so.

| |
|---|
| |

2. Clear the graph and search for data regarding laboratory measurements. Please explore this graph using your medical knowledge and verbalize your thoughts as you do so.

| |
|---|
| |

## Interview – Feedback Questions 3 (F)

1. On a scale of 1(very bad) to 10 (very good), how would you rate the user-friendliness of GraphXplore?

2. Which features or aspects of the tool do you find most useful?

3. What specific advantages do you see in this tool compared to other data exploration tools you know?

4. What specific disadvantages do you see in this tool compared to other data exploration tools you know?
