# Supplementary figures and images for: Introducing Attribute Association Graphs to Facilitate Medical Data Exploration: Development and Evaluation Using Epidemiological Study Data
Source: JMIR Med Inform. 2024 Jul 24;12:e49865. doi: 10.2196/49865 (PMC11306949; doi:10.2196/49865)

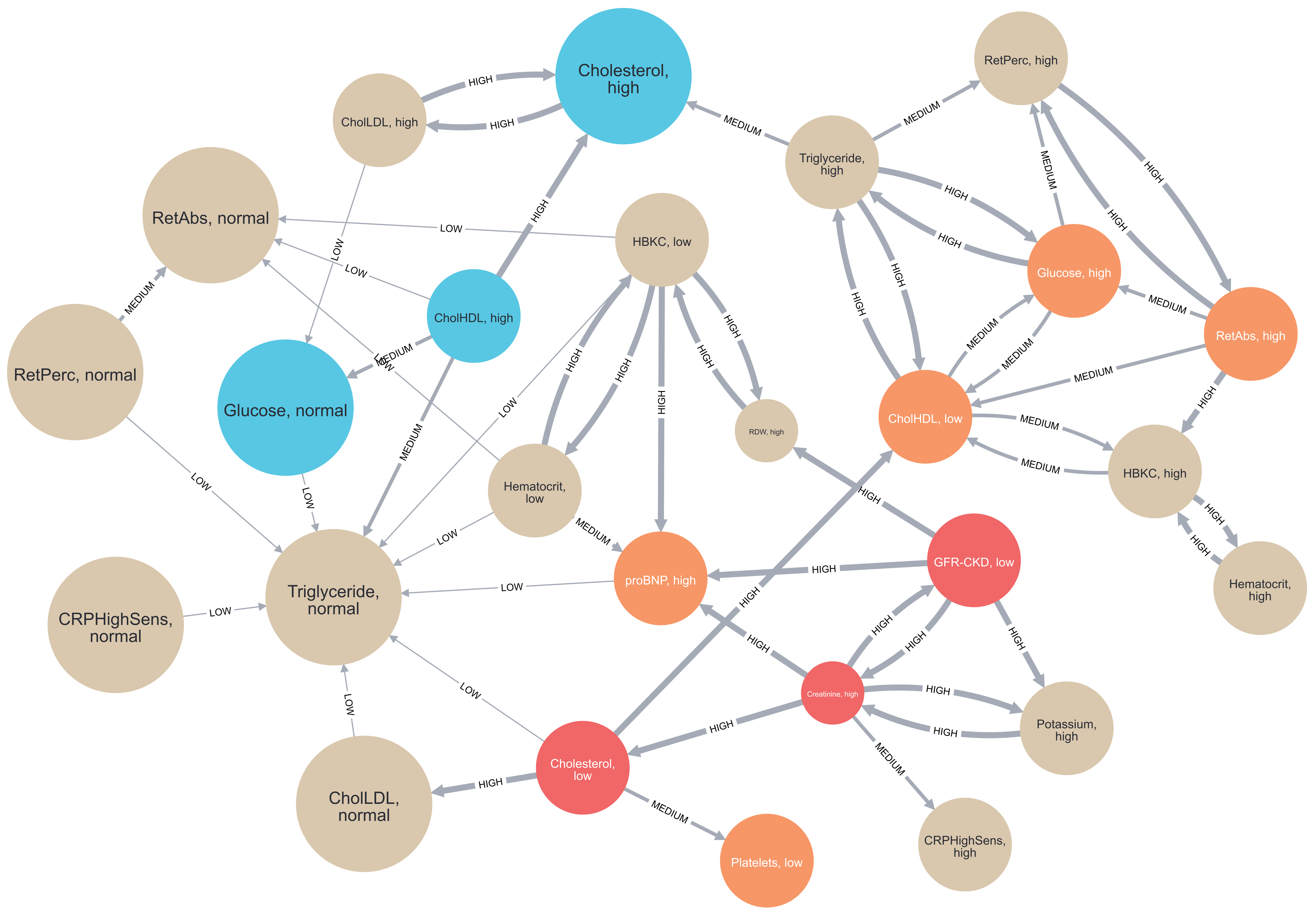

Supplement: Multimedia Appendix 5 [file medinform_v12i1e49865_app5.png]
